# Can “Stop The Bleed” training courses for laypersons improve hemorrhage control knowledge, skills, and attitudes? A systematic review

**DOI:** 10.1007/s00068-023-02422-6

**Published:** 2024-02-14

**Authors:** Rafael Consunji, Ahammed Mekkodathil, Husham Abdelrahman, Ayman El-Menyar, Ruben Peralta, Sandro Rizoli, Hassan Al-Thani

**Affiliations:** 1 Department of Surgery, Trauma Surgery, Injury Prevention, Hamad Medical Corporation (HMC), P.O. Box 3050, Doha, Qatar; 2 Department of Surgery, Clinical Research, Trauma & Vascular Surgery, HMC, Doha, Qatar; 3 Department of Clinical Medicine, Weill Cornell Medical College, Doha, Qatar; 4 Department of Surgery, Trauma Surgery, HMC, Doha, Qatar; 5 Department of Surgery, Universidad Nacional Pedro Henriquez Urena, 10100, Santo Domingo, Dominican Republic

**Keywords:** Stop the bleed, Trauma, Injury, Training, Course, Causality, Layperson, Review

## Abstract

**Background:**

In many regions of the world, most trauma deaths occur within 1–2 h of injury due to uncontrolled bleeding. For this reason, training lay first-person responders in trauma care, focusing on hemorrhage control, has been recommended. We hypothesized that STOP THE BLEED (STB) training courses that teach laypersons how to stop traumatic compressible bleeding immediately are needed to potentially prevent deaths due to hemorrhage. This systematic review will analyze the effect of the STB training course on the knowledge, skill, and attitudes of lay first-person responders for hemorrhage control.

**Methods:**

PubMed and Google Scholar databases were used to identify relevant peer-reviewed research articles describing evaluations of STB courses for laypersons from December 1 2013 to October 31 2022. In addition, a hand search of article references was undertaken. Studies were included if they implemented the STB course; trainees were laypersons, and the study had some outcome measures such as knowledge, skill, confidence gained, and willingness to provide or utilization of care provided to and outcomes of trauma patients.

**Results:**

The database searches yielded 2,893 unique papers. We retained 33 articles for full-text review, resulting in 24 eligible papers. Gray literature and manual searches yielded 11 additional publications for a total of 35 studies. The most reported finding was a statistically significant increase in hemorrhage control knowledge or tourniquet application skills in 26 studies. Twenty-two studies reported statistically significant improvements in willingness, confidence, comfort, and likelihood to respond to a bleeding patient, and 6 studies reported substantial reductions in the retention of bleeding control knowledge or skills. Only one study reported on the effect on patient outcomes.

**Conclusion:**

STB courses for laypersons have demonstrated significant improvements in knowledge, skill, confidence, and willingness to intervene to stop traumatic exsanguination. The evaluation of clinically relevant patient outcomes, specifically their effect on preventable deaths from traumatic exsanguination, is needed to strengthen further the evidence behind the recommendations for more widespread teaching of “STB” courses.

## Introduction

Trauma is the leading cause of death for the young and productive populations of the world; it is estimated that there are over 5 million deaths yearly from injuries [1]. Over 90% of these occur in resource-challenged, low, and middle-income countries [LMICs] [1].

The temporal death pattern for trauma is classically described as trimodal, and even after a more recent 'transition' to a bimodal model, most preventable trauma deaths still occur during the first peak, within hours of injury. This observation highlights the critical time following the injury, the classical 'golden hour,'' when timely, proven, and effective interventions may mean the difference between survival and certain death.

These early deaths are overwhelmingly due to traumatic exsanguination or uncontrolled bleeding [2]. These fatalities have been significantly reduced in settings with full trauma systems, most found in high-income countries [HICs]. The rapid transport of trauma victims to specialized trauma centers, where immediate hemorrhage control [HC] can be applied, has been identified as one factor that led to this improvement in trauma outcomes [3]. In the prehospital setting or at the scene, it must be noted that only compressible hemorrhage, often found in accessible sites, such as the extremities, can be addressed without sophisticated equipment and highly-trained personnel [4–9]. In support, the World Health Organization has identified the creation and/or improvement of prehospital trauma care as a priority for healthcare planners, especially in LMICs [5]. Furthermore, training lay first-person responders [LFPRs] in trauma care, with a focus on bleeding control for compressible hemorrhage, has been a recommended approach [5].

In the USA, the military experience from Iraq and Afghanistan informed recognition of the lifesaving potential of tourniquets to control bleeding immediately at the scene of injury. This practice was adapted and translated into the Tactical Combat Casualty Care (TCCC) course of the US military. The Bleeding Control Basic (B-CON) course, released to the public in 2014, developed and applied a curriculum focused on bleeding control, such as how cardiopulmonary resuscitation (CPR) prepares bystanders for a cardiac emergency. This, and the Hartford Consensus, influenced the creation and widespread implementation of the “STOP THE BLEED” course [STB] starting in October 2015 [8]. STB is the product of a collaborative effort led by the American College of Surgeons Committee on Trauma (ACS COT) to bring knowledge of bleeding control to the public. The ACS Committee on Trauma first publicly introduced bleeding control training courses for its members in October 2016. Since then, thousands of other medical professionals have trained to become course instructors [7]. Today, those instructors are focused on training laypeople from all walks of life to become immediate responders through the “STB” course [7]. In conjunction with these initiatives, a greater emphasis must be placed on evaluating these STB courses and implementing effective strategies for disseminating this crucial information to the public.

STB courses have these basic components: short didactic training followed by demonstrations, return demonstrations, and competency-based end-of-course evaluations. Some versions are conducted face-to-face, and there are online options, too. The rich literature on bystander training in CPR has shown that these courses improve the knowledge, skills, and attitudes of LFPRs and patient outcomes. It is, therefore, of utmost importance to create a similar evidence base upon which recommendations for the future implementation, widespread dissemination, and continued evaluation of STB courses can be built [6–9].

This systematic review analyzes the effect of the “STOP THE BLEED” training course on the knowledge, skill, and attitudes of lay first-person responders. We hypothesized that STB training courses are needed to improve the awareness, willingness, and skills in the community, which could subsequently reduce the burden of trauma.

## Methods

PubMed and Google Scholar databases were used to identify relevant peer-reviewed research articles describing evaluations of the STB courses for laypersons. In addition, a hand search of article references was undertaken. Studies were included if it implemented the STB course; trainees were laypersons without advanced medical, emergency medicine, or surgical training or experience in bleeding control, including but not limited to prehospital providers, community health workers, drivers, police, medical students, or laypersons; the study included some type of outcome measures such as knowledge, skill, confidence gained, willingness to provide or utilization of care provided to and outcomes of trauma patients. Studies were excluded if the training course was not the STB course; trainees were trained medical personnel with previous experience or training in trauma care; provided no details of training methods or details of the structure of the education; or if a subjective course evaluation or nontechnical skill evaluation (such as teamwork) was the only measurement tool used.

We developed, registered, and published a systematic review protocol (PROSPERO**;** CRD 42022302973) based on the Preferred Reporting Items for Systematic Reviews and Meta-Analyses) Statement (PRISMA).

## Information sources and searches

PubMed/MEDLINE database was queried to identify relevant peer-reviewed studies describing or evaluating the training of laypersons in [traumatic] hemorrhage control or simply trauma education/training programs from December 1, 2013 to October 31, 2022. Search terms used for PubMed/MEDLINE search were Stop the Bleed AND (Trauma) OR (Emergency) AND (Education) OR (Training) AND (Community) OR (layman/Layperson) OR Lay First Responder Trauma AND bleeding AND course OR training AND Skill OR (knowledge) OR (outcome) AND (non-specialist/layperson) OR Lay first responder. There were 123 results, 26 were reviewed, and 14 studies were selected.

A Google Scholar search was performed as a secondary search using the same search terms. There were 2,770 results, 27 were reviewed 16 were selected, and six were identified as redundant studies that had been previously chosen.

Only English-language articles were included for analysis. In addition, bibliographies of reviewed publications were crosschecked for additional relevant studies; this yielded 11 more studies for a total of 35 studies included in this systematic review.

## Eligibility criteria

### Inclusion

#### Population

Laypersons in the present review include the following but are not limited to,

- Community members.
- Teachers and other school employees.
- School or college students, including medical and nursing students.
- Law enforcement officers and security professionals.
- Commercial vehicle drivers.

#### Setting

- War or conflict.
- 'Daily' or routine trauma.
- Mass Casualty Incident.

#### Intervention

- Stop The Bleed education/training programs for laypersons.

#### Control

- Not required.

#### Outcome

- Acquisition of new knowledge and retention of existing knowledge [measured through test scores].
- Adequacy and retention of hemorrhage control skills [assessed by timing, proper application of tourniquets, or wound packing].
- Affective outcomes, including confidence, comfort, and/or willingness to perform hemorrhage control.

#### Study types

- Observational studies with pre/post design.
- Observational studies.
- Follow-up surveys.
- Randomized control trials.

#### Year of publication

- Published in the duration between January 1, 2013 and October 31, 2022.

#### Language

- English.

### Exclusion

- The study population includes only military persons.
- The study population comprises only healthcare professionals (physicians, nurses, EMS, pharmacists).
- Not original studies (books and documents, reviews, commentaries, letters, news articles, personal narratives, patient education handouts, case reports, legislation, and retracted publications).
- Studies evaluating specific equipment or teaching /educational techniques only.
- Studies reporting only the epidemiology of trauma victims.
- Psychological studies.
- Pilot studies.
- Non-English articles.
- Articles without full texts.

## Study selection

Two independent reviewers screened the titles and abstracts for inclusion in the full-text screening phase following the completion of the initial searches. Subsequently, in-depth evaluations of each full-text article were performed by two independent reviewers for inclusion in the synthesis and review. In the event of a disagreement, a third reviewer independently assessed all titles, abstracts, and full-text articles and served as a decision-maker.

**Quality assessment**: Due to the wide variations and heterogeneity of the available studies, we opted for the consensus of the four reviewers on the appropriateness and inclusion of the studies.

## Data extraction

For each included paper, two investigators independently extracted information on the study objective, study design, population, details about the intervention and control groups (mode and duration of education; emergency health conditions treated; and role of the layperson), outcomes (type of health outcome; description of health outcome; kind of emergency care provided; and effect size and confidence interval) and key conclusions. Where multiple publications were reported on the same dataset, we extracted data from all related papers and reported results from the most definitive paper.

We performed independent and duplicate assessments of study quality, including internal and external validity, selection and measurement biases, and confounding factors. We resolved discrepancies through consensus among the lead investigators.

### Data collection process

Data were extracted utilizing standardized forms with preset variables. This process was duplicated, with the authors mitigating any discrepancies for quality assurance.

### Data items

Data items included: first author, year published, country, study population, study objective, evaluation method, study design, sample size, and key findings.

### Synthesis

**Fig. 1:**
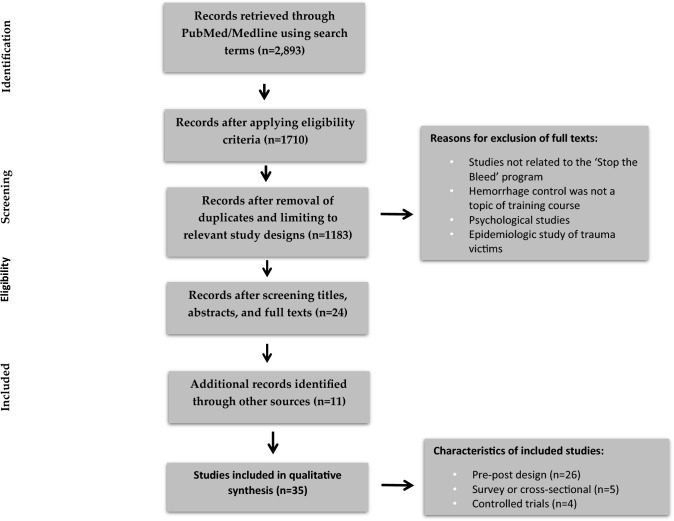
Flow chart on selection of articles for review

We prepared a summative and tabular synthesis of this review (Fig. 1). We tabulated each study's year of publication, location of study, study population, population size, study objective/s, study design, and outcome/s measured, including but not limited to knowledge, skill, willingness to perform bleeding control, confidence, retention of knowledge/skill and patient-related outcome/s.

## Results

The database searches yielded 2,893 unique papers. We retained 33 papers for full-text review, resulting in 24 eligible papers. Gray literature and manual searches yielded 11 additional publications, for a total of 35 articles in this systematic review.

### Study characteristics and STB course evaluations

Table 1 summarizes the studies that met the inclusion criteria and classifies the main findings of each of the studies based on the described outcome. There were 35 studies that evaluated the outcomes of the STB training course for laypersons [10–44]. Twelve were published in 2020 and 11 in 2019, 5 in 2021, 4 in 2018, and 3 in 2022. Thirty-one studies were conducted in the USA [89%] and 1 each in Colombia, Japan, Kuwait, and Sierra Leone. The target population of the courses was laypersons in 26 studies, medical students in 6, and stadium personnel in 3. Sample sizes ranged from 10 laypersons to 4,324 LFRs; the total study population, from 36 studies, was 15,406. The most commonly used evaluation tool was the pre and post-test in 33 studies; 4 added a retention evaluation, while 1 added a cost analysis to a post-course survey. There were two randomized controlled trials, one of which also added a retention evaluation. Ten studies evaluated willingness, confidence, comfort, and likelihood [WCCL] to respond to a bleeding patient as their primary objective. Thirteen aimed to assess knowledge acquisition, and 8 assessed tourniquet application skills after the course. Four declared the evaluation of knowledge retention as their primary aim. One sought to examine the frequency and effectiveness of STB interventions and reported the effect of the STB course on patient-related outcomes, i.e., encounters with bleeding patients and their survival after an STB intervention.

**Table 1:** Studies evaluating the stop the bleed (STB) programs in different population and settings

	Author (year) location,	Target population (no.), evaluation tool	Study objective	Knowledge/skill	Willingness, confidence, and comfort to intervene	Retention	Comments	Conclusions
1	Al Sabah (2018) Kuwait City, Kuwait [10]	Fifth year medical students (*n* = 150 from 1531 trained) Pre-posttests	To assess comprehension of trainees to demonstrate dealing with unstoppable bleeding, placing a tourniquet, respond to epistaxis, and recognize signs of internal bleeding	Pass rate [70%]; 94% improvement (from 2 to 96%)				STB improves bleeding control in trauma-naïve medical students
2	Goralnick (2018) Massachusetts, USA [11]	Stadium employees (*n* = 465 participants randomized to 4 arms) RCT	To assess correct application of a tourniquet in each study arm and at retention testing All participants received STB training to later assess retention	Bleeding control training (88% correct application) was superior to control (16%) 72% improvement		Only 54.5% retain this skill after 3 to 9 months; 33.5% decay	Adults aged 18 to 35 years and aged 35 to 55 years were associated with correct application at retention than those older than 55 years	Laypersons can successfully perform tourniquet application after undergoing a 1-h course
3	Pasley (2018) Maryland, USA [12]	Nonmedical, civilian personnel without prior tourniquet training (*n* = 10) Pre-posttests	To determine the retention of short-term knowledge and ability to apply a Combat Application Tourniquet (CAT)	Sixty percent were successful at tourniquet placement	Increased confidence in tourniquet application from 2.4 pretraining to 4.7 post-training	At 30 days, confidence decreased to 3.4 before testing		There is a significant reduction in confidence in applying the CAT 30 days after STB
4	Ross (2018) Texas, USA [13]	Laypersons (*n* = 218) Pre-posttests	To determine the impact of a brief hemorrhage control educational curriculum on the willingness of laypersons to respond during a traumatic emergency	12% statistically significant improvement in basic tourniquet knowledge with an initial score of 4.1/5 increasing to 4.7/5 (*P* < .001) post training. 10% statistically significant improvement in knowledge on where to place tourniquets with participants correctly identifying 3.1/4 correct placements at baseline and 3.6/4 (*P* < .001) following training	Willingness to use tourniquets in real life improved from 64.2 to 95.6% post-training. 31.4% improvement			The STB significantly improved knowledge on tourniquet placement and willingness to use them
5	Lei (2019) Texas, USA [14]	School nurses, medical students, researchers, and community members (*n* = 604) Pre-post training questionnaires	To assess participants' willingness & preparedness to intervene in a casualty event and knowledge of trauma/hemorrhage control	Improvement in knowledge regarding bleeding control techniques	A significant change in both *willingness* and *preparedness* after receiving training (*p* < 0.05)		Knowledge of the use of tourniquets and hemostatic agents improved after training	STB training is an effective tool to build confidence in participant's willingness and preparedness to respond to a stranger with severe bleeding
6	Jones (2019) Alabama, USA [15]	K-12 school personnel (*n* = 466) Cross‐sectional observational study	To evaluate STB training among K-12 personnel and assess participants’ perceived readiness to train peers in STB methods	Results revealed increased knowledge (4 [IQR 2–4] vs. 2 [1–2], *p* < .001) and comfort with (4 [2–5] vs. 2 [1–2], *p* < .001) STB skills	Participants felt more empowered to organize STB training (4 [3–5] vs. 3 [2–4], *p* < .001); those who felt empowered to organize STB training were eight times more likely to feel capable of teaching STB			STB increased Both knowledge and empowerment among K12 staff
7	Levy-Carrick (2019), Massachusetts, USA [16]	126 Medical School students (*n* = 126) Pre-, mid-, and post-training questionnaires	To examine the impact of a hemorrhage control training program on resilience-associated traits (role-clarity, self-efficacy, and empowerment) in medical students Examine the differential impact of additional hands-on skills training	There was a significant increase at each stage of training in self-reported role clarity about when to apply hemorrhage control skills (*p* < 0.01) and when not to apply them (*p* < 0.01)	Confidence in application of the skill (*p* < 0.01); as well as empowerment to apply the skill when appropriate (*p* < 0.01)			STB increased both knowledge and confidence of medical students
8	McCarty (2019) Boston, Massachusetts, USA [17]	Laypeople who attended a STB course (*n* = 102) Nonblinded, crossover, sequential randomized clinical trial with internal control	To assess whether participants completing the ACS (STB) training with CATs can effectively apply bleeding control principles using other tourniquet types (commercial and improvised)	Participants correctly applied the CAT at a significantly higher rate (92.2%) than all other commercial tourniquet types (Special Operation Forces Tactical Tourniquet, 68.6%; Stretch-Wrap-and-Tuck Tourniquet, 11.8%; Rapid Application Tourniquet System, 11.8%) and the improvised tourniquet (32.4%) (*P* < .001 for each pairwise comparison)				Consistent tourniquet type, in training and in application, leads to more consistent tourniquet application
9	Latuska (2019) Pennsylvania, USA [18]	School nurses (*n* = 16) Pre-post assessments	To assess the efficacy of a lecture-style STB seminar on improving school nurses’ knowledge related to bleeding control basics, competence in packing a wound and applying a tourniquet, self-confidence, and belief in school preparedness	Increase in knowledge in pre-post assessment (*p* ≤ .05). Increases in identification of characteristics of life-threatening bleeding in pre-post assessment (*p* ≤ .05). Hands-on skills competency increased following training (*p* ≤ .05)	Results demonstrated significant increases in both self-confidence and nurses’ perceptions of school preparedness following the STB training (*p* ≤ .05)			The STB training was effective in improving school nurses’ basic knowledge about life-threatening bleeding control and improved tourniquet application and wound-packing skills
10	Zwislewski (2019) Pennsylvania, USA [19]	Non-medical potential first responders [PFRs] (*n* = 298) Pre-post tests	To evaluate the efficacy of STB training for laypersons on knowledge and skill-based abilities in the workplace setting	PFRs scored higher in knowledge-based post-test (M = 4.63, SD = 1.32) than on the pre-test (M = 3.21, SD = 1.14). PFRs in the experimental group scored significantly higher (M = 7.41, SD = .91) than PFRs in the control group (M = 5.99, SD = 1.81) for tourniquet application Knowledge related to hemorrhage control increased following the STB course			Following the lecture, participants were divided into experimental and control groups during which hands-on practice was manipulated to determine the impact of guided practice on wound packing and tourniquet application	Participants who engaged in hands-on practice for tourniquet and wound packing were more proficient than those who only attended the lecture
11	Schroll (2019) Louisiana, USA [20]	Medical students (*n* = 423) pre-post evaluation of course	To assess the efficacy of medical student course participation as both learners and instructors; confidence in 6 major skill areas evaluated using a 5-point Likert scale	72.4% of medical students achieved perfect scores on their skill proficiency assessments	Post-course participant confidence increased significantly in all 6 core areas, including confidence to teach hemorrhage control skills to others			STB, taught by medical students, significantly increased their skill proficiency assessments and self-confidence. Medical students should be trained to increase the availability and reach of the STB program
12	Motameni (2019) Kentucky, USA [21]	Students (*n* = 4,324), and people from occupations fit broadly into first responders (n 483), health-care workers (n 424), school employees (n 449), office workers (n 461), and manual laborers (n 430). Survey (n = 571)	To identify populations most likely to benefit and performed a cost-analysis To provide an estimate of dollars spent and time required for each STB class	Majority of respondents found the lecture and the simulation helpful (*n* = 4558; 97.72% and *n* = 547; 95.80%, respectively)	Participants were feeling prepared to apply a tourniquet (*n* = 4 529; 92.64%) and feeling prepared to pack a bleeding wound (*n* = 4 510; 89.32%). Almost 99% stated they would recommend the course to others		Significant differences among groups who had previously been in situations where the STB course would have been helpful (88% first responders versus 40% students versus 43% public workers) (*P* < 0.001)	Focusing on specific high-yield groups rather than mass education might be a more efficient approach to STB education The average cost to host a class of 25 trainees was $729. $29.16 per trainee
13	Liu (2019) New York, USA [22]	Laypersons (*n* = 345, 43 STB training sessions) Pre-posttests	Nurse‑led teams to present course materials to people with no healthcare background and to evaluate the impact of this training by participants’ perceived ability to respond to life‑threatening bleeding		73% of participants reported increased confidence in responding to an emergency medical situation post-training, and 86% reported increased confidence in their ability to stop bleeding		95% of STB trainees reported feeling somewhat or extremely comfortable teaching newly acquired STB skills to someone else	Nurses should be trained to increase the availability and reach of the STB program
14	Chaudhary (2019) Massachusetts, USA [23]	Stadium employees (security, parking, food/beverage, and stadium operations) (*n* = 562) Pre-posttests	To evaluate the implementation of STB training at a stadium as a prospective model for general mass gathering site implementation To evaluate the retention of skills of participants 3 to 9 months after training	Only 16.4% were able to correctly apply tourniquet before the training, and 87.7% correctly applied after training	Increased mean likelihood to help (4.39 vs 4.09, *P* < .01) and comfort level to control hemorrhage (4.26 vs 3.60, *P* < .01) after training compared with before training, on a Likert scale (1–5)	55% of participants were able to successfully complete tourniquet application at retention testing	After the initial decay in tourniquet application skills, up to 3 months after the intervention, no significant further skill decay was noted	STB significantly increased the ability to correctly apply a tourniquet to stop bleeding, and likelihood to help and control hemorrhage. Refresher in 3 months
15	McCarty (2019) Maryland, USA [24]	Stadium employees (*n* = 317) Pre-posttests	To assess the effectiveness of laypeople with self-reported prior First Aid (FA) or Hemorrhage Control (HC) training with a tourniquet	Compared to participants with no prior training (14.4%, *n* = 16/111), those with FA training only (25.2%, *n* = 35/139) had a 2.12-higher odds (95% CI 1.07–4.18) of correct tourniquet application while those with FA + HC (35.8%, *n* = 24/67) had a 3.50-higher odds (95% CI 1.59–7.72) of correct application	Participants with prior FA + HC were more willing-to-assist and comfortable performing HC than those without prior training (*p* < 0.05). However, reporting being very willing-to-assist [OR 0.83, 95% CI 0.43–1.60] or very comfortable [OR 1.11, 95% CI 0.55–2.25] was not associated with correct tourniquet application		Self-reported prior FA + HC training, while associated with increased likelihood to correctly apply a tourniquet, results in only 1/3 of individuals correctly performing the skill	Study findings highlight the importance of effective layperson education techniques
16	Andrade (2020) Missouri, USA [25]	Community members (CM) and medical professionals (MP) (*n* = 80 CM and *n* = 60 MP) Pre- and post-course surveys	To determine if receiving a trauma first aid (TFA) kit in addition to Bleeding Control (BC) 1.0 training improves self-reported confidence among the participants		Both groups demonstrated improved confidence in their ability to stop severe bleeding after the training; however, post-class confidence was significantly modified by receiving a TFA kit	After training, CM confidence was 36.1% without versus 57.0% with a TFA kit (*p* < 0.05)		Receiving a TFA kit was significantly associated with increased post-training confidence among CM and MP
17	Bobko (2020) California, USA [26]	Nursing graduates & students, teachers, employees, security personnel, and high school students (*n* = 51)	To evaluate training a community population in access to wounded, recognition of injury, and rapid evacuation along with hemorrhage Control	At the arterial bleed simulation, the time elapse until their first action (T1A) for the trained and untrained groups, respectively, were 34.75 s and 111 s (*p* value = 0.1064), while the time to their solution of the simulation (TtS) were 3 min and 33 s in the trained group and 8 min in the untrained groups (physiologic cutoff) (*p* value = 0.0014)			Results from the comparison of T1A and TtS. between trained and untrained mirrored EMS times	STB significantly decreased the time elapse until their T1A and TtS to stop someone from bleeding
18	Ciraulo (2020) Maine, USA [27]	Educators and school support staff (*n* = 324) Pre-post evaluations	To train life- saving skills aimed at hemorrhage control and to evaluate improvement in readiness of school systems to respond in mass casualty incidents		Students demonstrated improvement post-course in all 7- questions relating to enhance confidence compared with the pre-course (*p* < 0.006)			STB significantly increased their confidence to stop someone from bleeding
19	Gowen (2020) Arkansas, USA [28]	Medical students (*n* = 96 surgery clerks) Pre-posttests	To study comfort and confidence level of students with all hemorrhage-control techniques	The mean percent correct on the post-training knowledge quiz was 96.7%	Significant increase in comfort and confidence among students with all hemorrhage-control techniques All students stated they would be willing to intervene in someone with life-threatening hemorrhage			STB significantly increased medical students’ knowledge, comfort, confidence and willingness to intervene in someone with life-threatening hemorrhage
20	Nanassy (2020) Pennsylvania, USA [29]	High School (HS) personnel (*n* = 156) Self-efficacy and school preparedness Likert scale survey	To assess the perceptions of self-efficacy and school preparedness in HS personnel		Perceptions of self-efficacy and school preparedness increased after the course [*p* < .001]			STB significantly increased self-efficacy and school prepared-ness to stop someone from bleeding
21	Orlas (2020) Cali, Colombia [30]	Allied health and non- allied health students Quasi-experimental study (*n* = 265) Pre-posttests	To evaluate the effectiveness of implementing the STB campaign and the association between the instructors’ background and the theoretical and practical competences achieved by the participants in Latin America. (Group A: surgeons; Group B: medical students)	Comparable post-training competence evaluations [theoretical test score]: among the groups Group A median, 5 (IQR 4–5); group B median, 5 (IQR 4–5); *P* = 0.41] and practical competency of tourniquet deployment group A (66.39%) versus group B (65.83%); *P* = 0.93] There was also no significant difference among both groups for correct tourniquet application [group A (66.39%) versus group B (65.83%); *P* = 0.93]	Willingness to intervene in bleeding control, increased from 92.2% without STB training to 99.4% after training (*P* < 0.001). Application of pressure to control bleeding increased from 84.21% before training increased to 98.68%) after training (*P* < 0.001), Significant increase in proportion to apply a tourniquet to a bleeding victim [pretraining: 43.42% versus post-training: 92.11%]	The likelihood to correctly apply pressure and apply a tourniquet increased significantly. Similarly, for participants with prior First Aid training with STB, the likelihood to use bleeding control skills increased after training		No significant differences between groups taught by surgeons and medical students in the post-training survey. Likelihood of taking action or applying a tourniquet increased after the course
22	Schroll (2020) Louisiana, USA [31]	Medical rescuers (MR) and lay rescuers (LRs) (*n* = 1,974) Pre-posttests	To evaluate the efficacy of STB training for rescuers from different backgrounds Students’ ability to perform STB skills using an internally validated 15-point objective assessment tool	Objective assessment of LR skills at the end of the course demonstrated a combined 99.3% proficiency on post course objective assessments	Post course confidence improved for both groups in all 6 main areas (*P* < 0.001). The most significant increases were reported in the two previous areas of lowest pre-course confidence-management of active severe bleeding and ability to pack a bleeding wound		STB training was effective, with both LRs and MRs demonstrating improved confidence and skill proficiency after a 1-h course. Building a pool of instructors, continued training of LRs, and determining how often skills should be recertified remain crucial	The STB improved confidence and skill proficiency after a 1-h STB course
23	Villegas (2020) New York, USA [32]	Hospital and community members (*n* = 310) Pre-posttests	To determine if STB training increased confidence and the likelihood of participants to respond to mass casualty; to determine if use of life-like mannequin would be beneficial; and whether these outcomes were attenuated by previous emergency training	Pre and post test score 24 of 30 (IQR 16–30). and 29 (IQR 25–30, *P* < 0.05) respectively	Participants reported that the training would be more effective if it were more realistic		Mannequins demonstrating pulsatile blood flow and cessation of hemorrhage could enable learners to actually “Stop the Bleed”	participants without prior experience or training in hemorrhage cessation demonstrated the most improvement
24	Muret-Wagstaff (2020) Georgia, USA [33]	College students with no previous trauma training (*n* = 30) Survey (*n* = 437)	To test the feasibility and effectiveness of an STB program enhanced by stepwise mastery learning with deliberate practice To learn the feasibility and effectiveness of an STB course using evidence-based procedural training techniques. A 3-point, behaviorally explicit checklist was used to test 4 skills: apply direct pressure; apply standard and improvised tourniquets; pack a wound	All participants reached mastery level for all 4 hemorrhage control skills within 4 tries. Additionally, 87% could state a definitive sign of life-threatening bleeding	76% predicted comfort using a tourniquet in a real-life emergency		Addressing the challenges of knowledge retention and skill decay, just-in-time innovations, implementation science methods to broaden access, and barriers to responding to real-life crisis events are important	The STB improved hemorrhage control skills and predicted comfort using a tourniquet in a real-life emergency after a 1-h STB course
25	Ito (2020) Tokyo, Japan [34]	14 physicians, 6 nurses, 82 medical students, 33 emergency services personnel, 22 police officers/security personnel (*n* = 157) Pre-post tests	To assess knowledge using pre and post-training tests comprising five questions	The mean ± SD scores pre and post training were 2.1 ± 1.1 and 3.2 ± 1. (*p* < 0.01). The lowest % of correct answers and the poorest improvement was on the actions which should be prioritized for bleeding victims			The STB program was valuable in preparing Japanese people for mass casualty events during the Tokyo Olympic & Paralympic Games in 2021	The STB program improved tourniquet knowledge. However, it was less effective in improving knowledge about which actions to prioritize for bleeding victims
26	Weinman (2020) New Jersey, USA [35]	Laypersons (*n* = 46) Pre-post tests	To determine to what degree the skill of commercial tourniquet [CTQ] application was retained 6 months after participation in an STB training session	30% participants attained a score of 100%, and 61% achieved a passing score. Bleeding was stopped or reduced to non–life-threatening levels by 74% The baseline score on the tourniquet skill test was 100% following initial training		At 6 months, 39% of participants were unable to successfully apply a tourniquet, and 26% were unable to control life-threatening bleeding At 6 months, mean scores were lower, 69% (*P* < 0.001)	Of the volunteers who failed, 18% stopped the bleeding, 18% slowed bleeding to a non–life threatening level, and 64% were unable to control bleeding Participants with passing scores were more likely to stop or reduce the bleeding than those with failing scores (97% vs 35%, *P* < 0.001)	The STB program improved tourniquet skill test scores. It also demonstrated that STB refresher training is needed within 6 months of initial training
27	Stadeli (2020) Seattle, Washington, USA [36]	Community participants (*n* = 27) Pre-post tests	To demonstrate feasibility and acceptability of a novel pilot program to deliver a culturally adapted STB curriculum for a cultural/ethnic minority and limited-English proficiency population	Bleeding control knowledge improved from 62 to 72%,	Self-efficacy to perform bleeding control skills improved from 65 to 93% pre-to-post survey		Most (96%, participants would recommend the training to a friend. Participant willingness to call EMS and to perform bleeding control was 100% on the pre and post surveys	WE STB was a feasible and acceptable program to increase bleeding control knowledge and self-efficacy among participants and build trust between participants and first responders
28	Ali (2021) New York, USA [37]	Private security and law enforcement personnel (*n* = 151) Pre-posttests	To evaluate the course by measuring improvements in tourniquet application skills and in trainees’ level of comfort with the procedure	Tourniquet was applied correctly by 17.2% and 92.7% Mean times to apply the tourniquet were 29.8 ± 18.5 and 18.7 ± 6.7 s, respectively at the pre- and post-instruction assessments	Subjects reported their level of comfort with the tourniquet pre-post to be 5.1 ± 3.3 and 8.8 ± 2.2, respectively (*p* < 0.001), and their familiarity with anatomy and bleeding control to be 5.2 ± 3.1 and 8.2 ± 2.4, respectively (*p* < 0.001)		At the end of the course, the mean score in response to a question about the extent to which the explanation had helped was 9.0 ± 1.9 (95% CI 8.7–9.4) and to a question about the extent to which teaching would make them feel more secure and safe was 9.2 ± 1.9 (95% CI 8.9–9.5)	STB course improved correct tourniquet placement, and demonstrated dramatic improvements in application time,
29	Moton (2021) Texas, USA [38]	Teachers and school staff (*n* = 437) Pre-post-tests	To evaluate the efficacy of pharmacist-led STB trainings for school employees in South Texas	Knowledge regarding tourniquets (70.86/100 vs 75.84/100; *P* < 0.0001) and proper tourniquet placement (2.40/4 vs 3.15/4; *P* < 0.0001) significantly improved	Compared with baseline, comfort level using tourniquets improved (mean, 3.17/5 vs 4.20/5; *P* < 0.0001), opinion regarding tourniquet safety (2.59/3 vs 2.94/3; *P* < 0.0001),		Trainees indicated this event was useful, as 96.6% rated it as either useful or very useful, with a mean rating of 4.9 of 5	Pharmacist-led STB trainings are efficacious in increasing school worker knowledge and willingness to respond in an emergency hemorrhagic situation
30	Jafri (2021) New York, USA [39]	School personnel (*n* = 66) Pre-posttests & follow-up survey	To compare standard STB vs. STB + simulation & feedback, and evaluation of retention of skills Retention testing was performed 2 to 8 months after intervention	The intervention group was 11.28 [*P* = 0.015, 95% CI = 1.86 to 104.67] times more likely to successfully apply a tourniquet on initial testing than the control group. The intervention group had a 90.8% correct tourniquet application rate while the rate in the control group was 71.0%	Augmenting STB with simulation and feedback improved both self-reported comfort level to perform bleeding control and wound packing	For retention assessment, it was demonstrated that the intervention group was 7.81 (*P* = 0.11; 95%CI = 0.70 to 196.42) times more likely than the control group to successfully apply a tourniquet, but not statistically significant		Augmenting STB with simulation and feedback improved both self-reported comfort level and skill set of participants, but the retention of skills was poor in both groups
31	Sainbayar (2021) Arizona, USA [40]	First year medical Students (*n* = 153) pre-posttests	To investigate whether intermittent STB reviews videos were effective for long term retention of hemorrhage control skills and improving perceived confidence	Two of the three knowledge-based quiz format questions significantly improved from pretraining to post-training (*p* < 0.001)	Intermittent review videos for STB training improved medical students’ confidence in their hemorrhage control skills		Videos did not improve their ability to correctly answer quiz-format questions compared with the control group	STB trainings are effective in increasing medical student knowledge and confidence
32	Goolsby (2021) Oregon, USA [41]	High school students from 39 states at a 2019 national conference (*n* = 248) RCT	To determine high-school students’ ability to learn hemorrhage control skills and knowledge via 3 educational modalities	Participants applied a tourniquet correctly: 88% instructor-led, 61% web-only, and 94% blended. The instructor-led and blended arms were superior to the web-only arm (*P* < .001). Nearly all participants passed an assessment requiring them to identify wounds warranting a tourniquet (99% instructor-led and blended, and 98% web-only)	All modalities Improved participants’ self-reported willingness and comfort in using tourniquets (*P* < .001)		study findings expand health educators’ ability to teach injury response to teens	High schoolers can learn the knowledge and skills to stop bleeding via multiple modalities
33	Okereke (2021) New York, USA [42]	Students from physical education or health education class (*n* = 286) pre-posttests	To test if students would have a greater degree of comfort, willingness, and preparedness to intervene in acute bleeding following the STB course		Pre and post proportion for somewhat likely or very likely to help an injured bleeding person were 43.8% and 80.8% respectively. Significant improvements in self-rated comfort level from pre- to post-course 45.4% to 76.5%, and in self-rated preparedness from 25.1% to 83.8%., (*P* < .0001 for all)			Students’ willingness to act could decrease pre-hospital blood loss and empower youth to perform life-saving interventions
34	Parvin-Nejad (2022) Kabala, Sierra Leone [43]	Nursing students (*n* = 121) One month and 1 year after the course, trainees completed follow-up surveys describing encounters with traumatic hemorrhage victims since the course	To examine the frequency and effectiveness of STB interventions by quantifying nursing student trainees’ encounters with bleeding victims after STB training in rural Sierra Leone			At the 1-month follow-up survey, with 75% reporting at least one encounter with a bleeding victim. This increased to 98% at 12 months (100 responses, average 2 ± 2 encounters)	Respondents intervened in 99% of encounters, and 97% of patients receiving intervention survived Although only 20% of respondents used a tourniquet, this technique produced the highest survival rate (100%)	These findings indicate the lifesaving impact of STB training in one rural Low and Middle Income (LMIC) setting
35	Petrone (2022) New York, USA [44]	Security personnel and civilians (*n* = 234) pre-posttests	To evaluate the efficacy and confidence level of security personnel placing a tourniquet compared to civilians		Statistically significant improvement between the pre- and post-training responses in both groups with respect to comfort level in placing a tourniquet		While civilians had the greatest increase in comfort level, the security personnel group had most significant reduction in the time to successful tourniquet placement	Participants improved their confidence level with the use of hemorrhage control techniques and dramatically increased the rate and reduced time to successful tourniquet placement

The most commonly reported finding was a statistically significant increase in hemorrhage control knowledge or tourniquet application skill in 26 studies. Twenty-two studies reported statistically significant improvements in WCCL to respond to a bleeding patient, and 6 studies reported on significant reductions in the retention of bleeding control knowledge or skills from 1–9 months after the course.

## Discussion

This systematic review of the literature found that the STB training course could improve hemorrhage control knowledge skills, confidence, comfort, and willingness to apply hemorrhagic techniques or equipment to a bleeding patient. These improvements are seen in locations in the USA and selected middle- to high-income countries. Another significant finding is that there is considerable decay in the knowledge and skills acquired between 1 and 9 months after these courses. A thorough evaluation of these courses' impact on patient-centered outcomes and in low-income countries must be conducted in the course of more widespread implementation of STB across the globe.

We applied a standard systematic review methodology for the significant period to assess four important outcomes from hemorrhage control training: the acquisition of knowledge and skill, the retention of both, and patient outcomes. This study built on earlier work by Callese et al. [45] focusing solely on training that only included the recently implemented STB courses and was limited to 'trauma-naïve' layperson first responder populations.

We acknowledge that we only reviewed published studies and that there is a clear publication bias inherent to our study design. We cannot dispute that few publish their failed experiments, but not all successes are trumpeted either. All lessons learned from this review are based on reports from 35 hemorrhage control papers with a total of 15,406 STB participants in various settings.

A further selection bias will be in the student selection for these courses. This bias will inevitably lead to response bias, especially for the affective outcomes. Despite this, there were still sub-populations of students who found the content would have been more helpful to them, i.e., first responders [20]. We considered the inter-study heterogeneity as “very high” in many ways, like the setting, faculty, students, methods, and evaluation tools, but thought of it as further proof of the consistency of the effect of the courses. It almost did not matter who the target population of these courses was, nor did it matter who composed the faculty [19, 29, 37]. The consistent and uniform effect was a significant improvement in skills and knowledge, as well as the willingness to provide a hemorrhage control intervention after the course was taken.

Callese et al. conducted a systematic review that examined existing educational initiatives for layperson first responders in LMICs to inform the design of prehospital trauma care systems in resource-poor settings [45]. The study period of this review was from 1965 to November 2013, and they included 13 manuscripts. Four themes emerged from this review:

1. An initial needs assessment of a region's existing trauma system of care and laypersons' baseline emergency care knowledge focuses on subsequent educational interventions.
2. Effective programs adapt to and leverage existing resources.
3. Training methods should anticipate participants with low levels of education and literacy.
4. Postimplementation evaluation allows for curriculum improvement. Technology, such as online and remote learning platforms, can be used to operationalize each theme [44, 46].

Furthermore, they concluded that for layperson first responders in LMICs, successful training programs identify and maximize existing resources that are adaptable to learners with little formal education and are responsive to post-implementation evaluation.

This review analyzed the effect of hemorrhagic control training on the knowledge, skills, and attitudes of lay first-person responders and the outcomes of patients to whom their hemostatic interventions were applied. Its study period began when Callese et al.[45] ended in December 2013, and it included studies from all of the locations where the STB course was implemented. We focused on the training or education of lay first responders because this is the target population of the STB course. The choice of knowledge, skills, and Willingness, Confidence Comfort towards hemorrhage control, as our primary outcomes, were based on the epidemiology of preventable prehospital or early trauma deaths; the majority expire from exsanguination and low levels of willingness amongst the general public, to intervene to control bleeding in emergency settings [46–50].

We acknowledge that most of the STB studies were more recent; the STB was only implemented in 2015, and 90% were performed in the USA. These limitations are inherent to the course's history, origins, and evolution. We excluded any equipment or device specific evaluation as we were more focused on an evaluation of bleeding control through cognitive, psychomotor, and affective elements, rather than on new tourniquet design [47]. In this same vein, we acknowledge that pre and post-test results are process evaluations that cannot replace objective measurements of bleeding control.This review may have overlooked some non-US-based STB evaluations as it did not review non-English publications and did not query additional, more internationally-focused, databases such as Embase, Cochrane Web-of-Science, and BIOSIS search engines. Furthermore, vital manuscripts could have been overlooked if only a title/abstract review was utilized. Finally, the authors did not follow a well-established grading score for the assessment of the study's quality apart from their consensus.

The fact that the STB course material is being made available, without charge or costs, by the ACS-COT makes this analysis even more compelling as it can serve as a basis for comparison to other non-STB bleeding control courses not only using traditional measures but also cost-effectiveness. However, the affordability of tourniquets or equivalent bleeding control equipment per se will undoubtedly influence the acquisition of such. In the post-course setting, developing strategies that enhance or incentivize access to these materials is imperative. These will favor better implementation of the of the program exactly as it is taught in the STB courses [48, 49].

Almost all studies reported an increase in the bleeding control knowledge of students, measured by the post-tests. However, in Massachusetts, the gain in willingness and comfort to apply bleeding control did not readily translate to improvements in proper tourniquet application [24]. Multiple studies reported that certain sub-populations had a higher 'yield,' as measured by the percentage increase in correct responses to post-test questions. These sub-populations included individuals with prior training or experience in hemorrhage control, those with a shorter time to successful tourniquet placement, and security personnel [32, 44]. Of the six studies that measured bleeding control knowledge retention, the range of knowledge retention was 39–72%; the tests were performed from 1 to 9 months after the initial training [10, 11, 22, 34, 38, 39].

Of the studies that evaluated skill acquisition, it was found that the course significantly improved post-course skill evaluation scores in nine [9–11, 22, 32, 34, 37, 38, 40]. Incorporating a hands-on component simulation [18] or a bleeding model [31] resulted in not only better skill at bleeding control but also reduced time to achieve correct tourniquet application [36].

While the merits of knowledge, skill, or attitude as measures of the effectiveness of a bleeding control course have been described and discussed elsewhere [51], in the end, an HC course must demonstrate that it can reduce the number of deaths from exsanguinating trauma patients. The lone study that showed the lifesaving impact of the STB course was not even conducted in the USA. Parvin-Nejad and co-authors described the experience of STB-trained nursing students in Sierra Leone, 98% of whom had an encounter with a bleeding patient at 12 months. Although only 20% used a tourniquet, almost all patients [97%] survived [43].

Given the lack of evidence to support the positive effect of the 'alphabet' courses on trauma outcomes, we thought it would be most pragmatic to include patient outcomes in the performance indicators of these HC courses. And this is why we recommend that all HC course evaluations include specific, clinically significant patient outcome measures.

Four studies evaluated the effect of instructor/faculty characteristics, and they did not find any significant differences in the post-test results of students trained by faculty from different backgrounds [19, 21, 29, 37]. From a task-shifting or force multiplier standpoint, this supports more 'train the trainer' possibilities and options that utilize lay, non-medical faculty to further spread valuable bleeding control knowledge and skills within various communities.

Five studies evaluated the effect of participant/student characteristics on the acquisition of HC knowledge and utilization of HC skills. They found that younger students, from 18 to 55 years, retained HC skills significantly better than more senior students aged > 55 years [10]. First responders who had previous exposure to situations needing HC felt substantially more prepared when compared to students and public workers [20, 23]. Trauma-naïve students, without prior experience or training in HC**,** had more improvement in post-test scores than other students [31]. In contrast, another paper identified law enforcement and civilian participants with better post-test scores than firefighters [33] and security personnel with reduced time to successful tourniquet placement [43].

Only one study addressed the cost-effectiveness of teaching HC courses to lay persons. The average cost to host a class of 25 trainees was $ 729.00 ( $ 29.16 per student) to achieve significant improvements in feeling prepared to apply a tourniquet and to pack a bleeding wound [20].

Geographically, this review reveals that there are marked regional gaps in the locations of published evaluations of HC courses. For this review, no studies were included from the South-East Asian region (SEAR) and the European region (EUR).

### Meaning of the study and mechanisms and implications for policymakers

There is a clear message from 35 studies that evaluated the STB courses for laypersons that trained over 15,000 students. The message is that STB courses can improve students' knowledge, skill, WCCL and the key patients’ outcomes when they implement newly acquired lessons and skills learned. The broader implementation of these courses must be recommended. Still, there must be consensus on evaluation tools, process and outcome indicators, and the priority regions/areas where this must happen.

Many previous authors have enumerated recommendations to improve the Stop The Bleed courses [52–54]. While it is beyond the scope of this paper to list and discuss each of these suggestions, we will mention the domains that warrant closer focus as the global HC community moves forward with the STB course:

- To inform the frequency and format of refresher courses, well-designed studies to assess the retention of knowledge and skills must be performed [47, 48].
- With the initiative for public education on tourniquet use, instructions should support victims or first-responder laypeople assuming no education has been received. Given these courses' low penetration, consider using Just In Time [JiT] instructions for tourniquet application [46, 48].
- At a minimum, all STB education programs should achieve certain objectives [55, 56].
  These objectives are strategically structured, commencing with a focus on the affective domain to motivate learners to take prompt actions in response to hemorrhagic emergencies. Subsequently, the programs should be designed to target the cognitive domain, imparting knowledge to learners for distinguishing between life-threatening and non-life-threatening bleeding. Finally, STB education programs must provide comprehensive instructions on the practical application of pressure, combining both cognitive and psychomotor elements.
- While learner reactions inform how training programs are designed, programs must collect data at all levels of the Kirkpatrick model. Behavioral and results outcomes through follow-up data collection with trainees will tell the community whether or not the lay public is utilizing STB training to implement potentially lifesaving interventions [57].
- Just as there has been a consensus among many groups through The Hartford Consensus and the more than 60 organizations that support Stop The Bleed, is it also time to make the curriculum and training more standardized? [58].
- The STB program overwhelmingly improves short-term confidence, but gaps in skill retention, data collection on patient outcomes, and settings that would benefit are identified [51].

## Unanswered questions and future research

At this stage, is there certainly enough evidence to make this recommendation for the more widespread implementation of these STB courses? All things considered, any bleeding control course must demonstrate that it reduces the death toll from preventable deaths due to uncontrolled traumatic exsanguination. In the interim, while we await the definitive data demonstrating the survival benefit of the STB courses, the best evidence from this review should suffice to inform the more targeted and focused STB course implementation and consistent evaluation [59]. The proper implementation of such training courses would be expected to improve the potential morbidity and mortality of the casualties’ victims. However, most of the published studies did not specify how to measure these outcomes, and therefore the current systematic review could not assess them. Like the CPR courses, the learning curves and guidelines will improve with time once STB training courses are implemented in the right place and time [60, 61].

## Conclusions

Hemorrhage control courses for laypersons have demonstrated significant improvements in knowledge, skill, confidence, and willingness to intervene to stop traumatic exsanguination. The implementation of these courses needs be accompanied by more universal, consistent, and objective evaluations of learning objectives and their effect on desired clinical outcomes that apply the numerous published consensus recommendations. The evaluation of clinically relevant patient outcomes, specifically their effect on preventable deaths from compressible hemorrhage, is needed to strengthen further the evidence behind the recommendations for the more widespread teaching of the STB courses.

## Data Availability

All data were presented in the manuscript and tables.
